# *Sergentomyia schwetzi* is not a competent vector for *Leishmania donovani* and other *Leishmania* species pathogenic to humans

**DOI:** 10.1186/1756-3305-6-186

**Published:** 2013-06-20

**Authors:** Jovana Sadlova, Vit Dvorak, Veronika Seblova, Alon Warburg, Jan Votypka, Petr Volf

**Affiliations:** 1 Department of Parasitology, Faculty of Science, Charles University, Vinicna 7, 128 44 Prague 2, Czech Republic; 2 Department of Microbiology & Molecular Genetics, The Institute for Medical Research Israel-Canada, The Kuvin Centre for the Study of Infectious & Tropical Diseases, The Hebrew University - Hadassah Medical School, The Hebrew University of Jerusalem, Jerusalem 91120, Israel

**Keywords:** Visceral leishmaniasis, Phlebotomine sand flies, *Phlebotomus*, *Sergentomyia*, Peritrophic matrix

## Abstract

**Background:**

Sand fly species of the genus *Sergentomyia* are proven vectors of reptilian *Leishmania* that are non-pathogenic to humans. However, a consideration of the role of *Sergentomyia* spp. in the circulation of mammalian leishmaniasis appears repeatedly in the literature and the possibility of *Leishmania* transmission to humans remains unclear. Here we studied the susceptibility of colonized *Sergentomyia schwetzi* to *Leishmania donovani* and two other *Leishmania* species pathogenic to humans: *L. infantum* and *L. major*.

**Methods:**

Females of laboratory-reared *S. schwetzi* were infected by cultured *Leishmania* spp. by feeding through a chicken membrane, dissected at different time intervals post bloodmeal and examined by light microscopy for the abundance and location of infections.

**Results:**

All three *Leishmania* species produced heavy late stage infections in *Lutzomyia longipalpis* or *Phlebotomus duboscqi* sand flies used as positive controls. In contrast, none of them completed their developmental cycle in *Sergentomyia* females; *Leishmania* promastigotes developed within the bloodmeal enclosed by the peritrophic matrix (PM) but were defecated together with the blood remnants, failing to establish a midgut infection. In *S. schwetzi*, the PM persisted significantly longer than in *L. longipalpis* and it was degraded almost simultaneously with defecation. Therefore, *Leishmania* transformation from procyclic to long nectomonad forms was delayed and parasites did not attach to the midgut epithelium.

**Conclusions:**

*Sergentomyia schwetzi* is refractory to human *Leishmania* species and the data indicate that the crucial aspect of the refractoriness is the relative timing of defecation versus PM degradation.

## Background

Visceral leishmaniasis (VL) caused by *Leishmania donovani* is a serious health problem in parts of the Indian subcontinent and in several East African countries, mainly Kenya, Ethiopia and Sudan. Three sand fly species, *P.* (*Larroussius*) *orientalis*, *P.* (*Synphlebotomus*) *martini*, and *P.* (*Synphlebotomus*) *celiae*, have been incriminated as vectors in East Africa (reviewed by [1]). *Phlebotomus martini* and *P. celiae* are associated with the presence of termite mounds, soil moisture and a prolonged wet season while *P. orientalis* prefers drier habitats and is the main man-biter in *Acacia-Balanites* forests in Sudan and Ethiopia [2-4]. It is the dominant vector in the VL endemic areas in Sudan (reviewed by [5]) and the probable vector in most VL foci in Ethiopia [6,7]. However, although being a predominant species in some VL foci in north and northwest Ethiopia, no natural infection was detected in hundreds of females of *P. orientalis* examined [6,8]. In addition, VL is also present in localities such as the Malakal urban area in Sudan, where *P. orientalis* or other proven vectors of *L. donovani* were not found [9]. Therefore, vector competence of other sand fly species found in endemic areas has been tested. Recently, *P. rodhaini* was implicated as a possible zoonotic vector of *L. donovani* in woodlands in eastern Sudan [10]. However, *P. rodhaini* is rather a rare species with a low man-biting rate while the prerequisite of a vector of human pathogens is that it is abundant in the disease endemic areas and display man-biting behaviour.

*Sergentomyia* spp. are widespread in Africa, tolerate various biotopes and environments and are by far the predominant sand flies in many African ecosystems [2,11,12]. Sand flies of this genus are proven vectors of reptile *Leishmania* species, non-pathogenic to humans, which were previously separated to the genus *Sauroleishmania*[13], however, following recent DNA sequence-based phylogenies they have been included back into the genus *Leishmania* (reviewed by [14])*.* The development of reptilian *Leishmania* spp. in vectors is usually hypopylarian (occurring in the hindgut) with transmission by predation (lizards feed on infected sand fly) and not by bite, although infections of oesophagus, pharynx and proboscis have been reported [15]. However, *Sergentomyia* species are not restricted to feeding on reptiles and at least some of them feed on humans and/or mammalian reservoirs of *Leishmania* pathogenic to humans. Therefore, they were suspected as vectors in some VL and cutaneous leishmaniasis (CL) foci where *Sergentomyia* spp. were abundant and found to harbor *Leishmania*[16] or significantly associated with leishmaniasis seroprevalence [12]. Additional support for the role of *Sergentomyia* spp. in transmission of mammalian *Leishmania* was provided by a study performed in a *L. major* focus in Baringo district, Kenya [17], where *P. duboscqi* was proven as a primary vector. *S. ingrami* females were found to be infected in comparatively high rates (about 1%). Moreover, *Leishmania* parasites isolated from dissected *S. ingrami* guts and inoculated into BALB/c mice caused typical *L. major* lesions; smears from lesions revealed numerous amastigotes. Therefore, *S. ingrami* was considered by the authors as a secondary zoonotic vector of *L. major* in the Baringo focus [17].

*Sergentomyia schwetzi* has a wide range of distribution in Africa, south of the Sahara. It predominated among all sand fly species caught in Senegal [12], southern Ethiopia [18] and eastern Sudan [11,19] showing strong endophilic behaviour [9,19] and man biting tendencies [7,9]. In a focus of VL in northern Ethiopia, *S. schwetzi* was exceptionally abundant and the only *Sergentomyia* species attracted to CO_2_ (Kirstein and Faiman, personal communication). In such setting, the apparent question emerges whether *S. schwetzi* could be incriminated in *Leishmania* transmission to humans and thus plays a role in the epidemiology of the disease. To test if *S. schwetzi* supports the full developmental cycle of *Leishmania* spp. that are pathogenic to humans, we experimentally infected laboratory-reared *S. schwetzi* with *L. donovani*, *L. infantum* and *L. major*. The permissive vector species *Lutzomyia longipalpis* and the proven vector of *L. major, P. duboscqi*, were chosen as positive controls.

## Methods

### *Leishmania* and sand flies

*Leishmania major* (MHOM/IL/81/Friedlin/VI; FVI) was cultured in M199 medium (Sigma) containing 10% heat-inactivated fetal calf serum (Gibco) and 250 μg/ml amikacin (Amikin, Bristol-Myers Squibb). *L. donovani* (MHOM/ET/2010/GR374) and *L. infantum* (ITOB/TR/2005/CUK3) were cultured in the same medium supplemented by 1% BME vitamins (Sigma) and 2% sterile urine. The colony of *S. schwetzi* was established from specimens collected in Sheraro (14° 24' 09.69''N – 37° 46' 39.69''E), a town in north-western Ethiopia, located in the Mi’irabawi Zone of the Tigray Region. Laboratory colonies of *L. longipalpis* (from Jacobina, Brazil) and *P. duboscqi* (from Senegal) served as a control. All three sand fly colonies were maintained at 26°C on 50% sucrose and 14 h light/10 h dark photoperiod as described previously [20].

### Sand fly infections

Female sand flies (5–9 days old) were infected by feeding through a chick-skin membrane on heat-inactivated rabbit blood containing 10^6^ promastigotes ml^-1^. If not stated otherwise, engorged sand flies were maintained in the same conditions as the colony. The effect of temperature was tested by comparison of parasite development at 21°C. Females were dissected at different time intervals post-bloodmeal (PBM), the abundance and location of *Leishmania* infections in the sand fly digestive tract were examined by light microscopy. Parasite loads were graded according to [21] as light (< 100 parasites per gut), moderate (100 to 1000 parasites per gut) and heavy (> 1000 parasites per gut). Experiments with each *Leishmania* – sand fly combination were repeated twice or three times.

### Morphometry of parasites

On day 2 post-bloodmeal midgut smears of *S. schwetzi* and *Lu. longipalpis* infected with *L. donovani* were fixed with methanol, stained with Giemsa, examined under the light microscope with an oil-immersion objective and photographed with an Olympus D70 camera. Body length, flagellar length and body width of 300 randomly selected promastigotes from five females/smears were measured for each sand fly species using Image-J software.

### Statistical analysis

Differences in intensities of infections, presence vs. absence of peritrophic matrix and remnants of blood were tested using Fisher’s exact test (for 2 × 2 contingency tables) or Chi-square tests. Measurements of parasites were compared using Analysis of variance. All the statistical evaluations were performed with statistical software SPSS v. 16.

## Results

### Development of three *Leishmania* species in *S. schwetzi*

**Figure 1:**
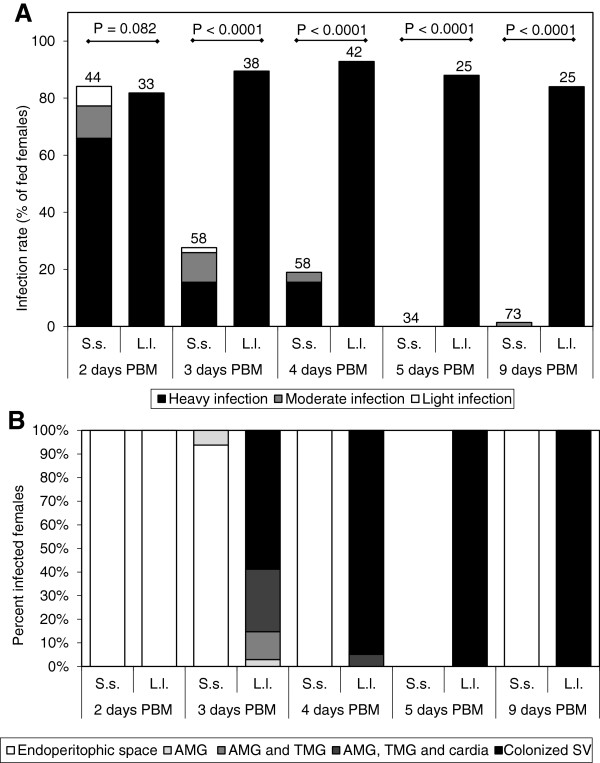
**Development of *L. donovani *in sand flies at 26°C. A)** Rates and intensities of infections in *Sergentomyia schwetzi* (S.s.) and *Lutzomyia longipalpis* (L.l.). Numbers of dissected females are shown above bars. Probability of differences was tested by Chi-square test. **B)** Location of *L. donovani* in infected *Sergentomyia schwetzi* (S.s.) and *Lutzomyia longipalpis* (L.l.). AMG, abdominal midgut; TMG, thoracic midgut; SV, stomodeal valve.

Development of *L. donovani* in *S. schwetzi* was followed from day 2 to 9 PBM and compared with development in *Lu. longipalpis*, sand fly known to be highly susceptible for this *Leishmania* sp. [22]. On day 2 PBM, heavy infections were enclosed inside the peritrophic matrix (PM) in most females of both species. However, further development differed considerably (Figure 1). In *L. longipalpis*, parasites developed heavy infection of the abdominal midgut (AMG) and thoracic midgut (TMG) and had started to colonize the stomodeal valve (SV) region by day 3 PBM already; infection rates did not fall below 80% throughout the experiment.

On the other hand, infection rates in *S. schwetzi* rapidly decreased to 28% by day 3 PBM, 19% by day 4 PBM and 1.4% by day 9 PBM. In all but one positive female (n = 65), parasites were located within the bloodmeal and enclosed by the intact PM. In a single female, promastigotes were observed swimming freely in the AMG but we cannot exclude the possibility that they were released due to a damage of the PM during dissection.

The morphology of *L. donovani* was studied on day 2 PBM when the bloodmeal was still enclosed inside the PM in 100% of *S. schwetzi* and 90.9% of *L. longipalpis*. Although both sand fly species were infected by the same parasite culture, the body length of *L. donovani* developing in *L. longipalpis* was significantly higher than that of parasites developing in *S. schwetzi* (Table 1).

**Table 1:** **Body length of *L. donovani *developing in sandflies**

	**N**	**Mean (S.D.) (μm)**	**Range (μm)**	**Significance of difference between vector species (ANOVA)**
*S. schwetzi*	300	9.24 (3.39)	3.5-17.9	F = 180.251; d.f. = 1, P < 0.0001
*L. longipalpis*	300	12.83 (3.15)	4.7-22.1	

Parasites were measured from blood smears of sand flies dissected by day 2 PBM.

**Figure 2:**
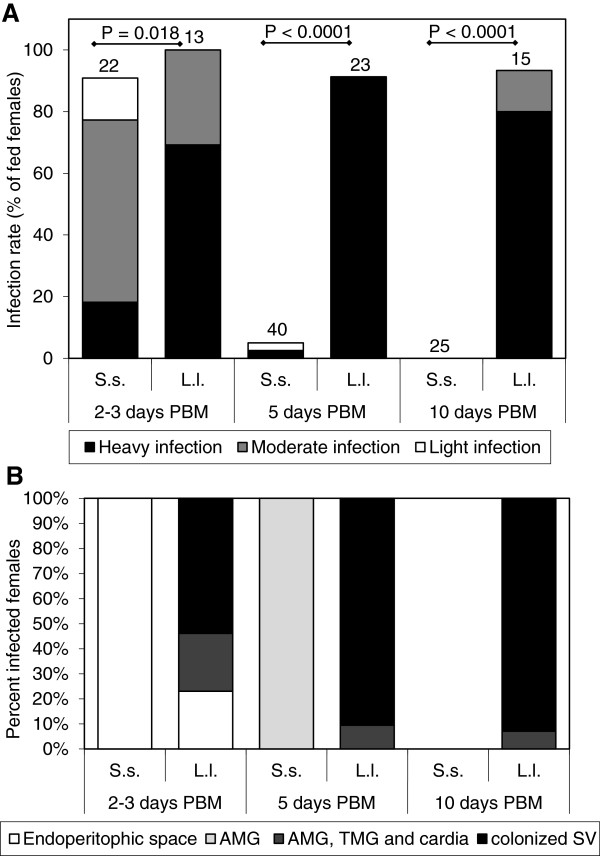
**Development of *L. infantum *in sand flies. A) **Rates and intensities of infections in *Sergentomyia schwetzi* (S.s.) and *Lutzomyia longipalpis* (L.l.). Numbers of dissected females are shown above bars. Probability of differences was tested by Chi-square test. **B)** Location of *L. infantum* in infected *Sergentomyia schwetzi* (S.s.) and *Lutzomyia longipalpis* (L.l.). AMG, abdominal midgut; TMG, thoracic midgut; SV, stomodeal valve.

**Figure 3:**
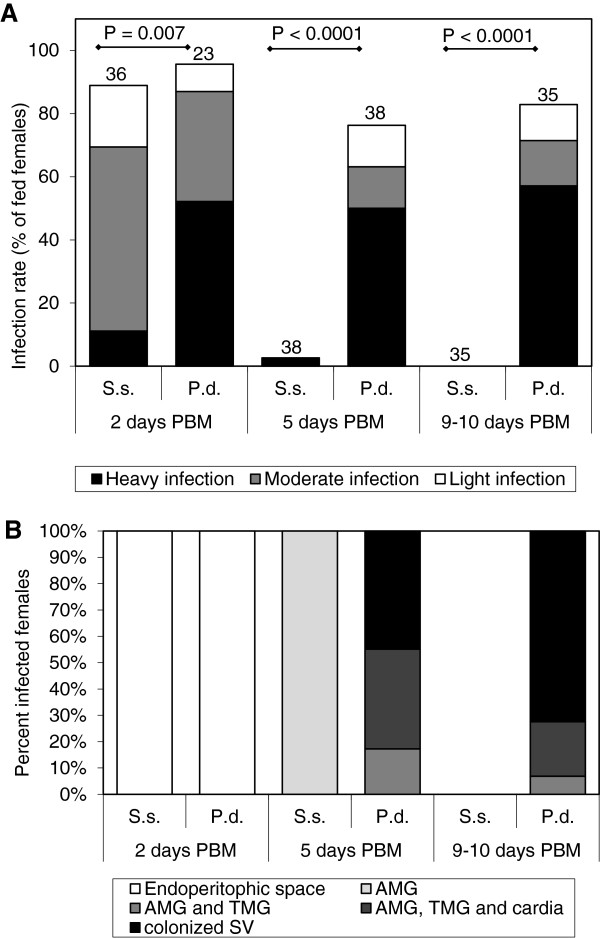
**Development of *L. major *in sand flies. A)** Rates and intensities of infections in *Sergentomyia schwetzi* (S.s.) and *Phlebotomus duboscqi* (P.d.). Numbers of dissected females are shown above bars. Probability of differences was tested by Chi-square test. **B)** Location of *L. major* in infected *Sergentomyia schwetzi* (S.s.) and *Phlebotomus duboscqi* (P.d.). AMG, abdominal midgut; TMG, thoracic midgut; SV, stomodeal valve.

Notably, like *L. donovani, L. infantum* and *L. major* infections did not thrive in *S. schwetzi* either (Figures 2 and 3). During early phases of infection, when parasites were still inside the endoperitrophic space, infection rates were comparable with those reached in control vectors, i.e. *L. longipalpis* and *P. duboscqi* with *L. infantum* and *L. major*, respectively. However, on day 5 PBM, only one *L. major* and two *L. infantum* infections were found in the abdominal midgut of *S. schwetzi* and no parasites survived till day 9 or 10 PBM.

To explain the different competences of *S. schwetzi* and *L. longipalpis* for *L. donovani*, we focused on physiological differences between these two sand fly species, namely the kinetics of the PM development and the defecation of bloodmeal remnants. We also tested development of *L. donovani* infections in females maintained under different ambient temperatures (see below).

### Kinetics of the development of the PM and the defecation of digested blood remnants

Table 2 shows highly significant interspecific differences in formation of the PM by days 3 and 4 PBM. While in *L. longipalpis* the PM was found to be present in 8% and 0% of sand flies on days 3 and 4 PBM, respectively, in *S. schwetzi* it persisted longer and was still present in more than 20% of females on day 4 PBM. The interspecific difference was even more pronounced in infected females (Table 2). Defecation of blood meal remnants was faster in *L. longipalpis*; by day 4 PBM all but one female of this species finished defecation (Table 2). Importantly, on day 3 PBM, the percentage of females in which the PM had already degraded but which had not yet defecated the blood remnants was significantly higher in *L. longipalpis* (Table 3).

**Table 2:** Presence of the peritrophic matrix (PM) in sand flies maintained at 26°C

**Day PBM**	**Sand fly species**	**All fed females**			**Females infected with *L. donovani***		
		**Females with PM present / Total N**	**Percent**	**Significance of interspecific difference (Fisher’s exact test)**	**Females with PM present / Total N**	**Percent**	**Significance of interspecific difference (Fisher’s exact test)**
2	*S. schwetzi*	44/44	100	P = 0.075	37/37	100	P = 0.422
	*L. longipalpis*	30/33	91		26/27	96	
3	*S. schwetzi*	17/58	29	P = 0.019	16/16	100	P < 0.001
	*L. longipalpis*	3/38	8		3/34	9	
4	*S. schwetzi*	12/58	21	P = 0.001	11/11	100	P < 0.001
	*L. longipalpis*	0/42	0		0/39	0	
5	*S. schwetzi*	0/34	0	Not computed	-	-	Not computed
	*L. longipalpis*	0/25	0		0/22	0	
9	*S. schwetzi*	1/73	1	P = 1.000	1/1	100	P = 0.045
	*L. longipalpis*	0/25	0		0/21	0	

**Table 3:** Blood defecation of sandflies maintained at 26°C

**Day PBM**	**Sand fly species**	**Females that finished defecation / Total N (%)**	**Significance of interspecific difference (Fisher’s exact test)**	**Females with the PM intact or slightly disintegrated / Females which did not defecate (%)**	**Females which the PM degraded / Females which did not defecate (%)**	**Significance of interspecific difference (Fisher’s exact test)**
2	*S. schwetzi*	0/44 (0)	Not computed	44/44 (100)	0/44 (0)	P = 0.075
	*L. longipalpis*	0/33 (0)		30/33 (91)	3/33 (9)	
3	*S. schwetzi*	37/58 (64)	P = 1.000	17/21 (81)	4/21 (19)	P = 0.001
	*L. longipalpis*	25/38 (66)		3/13 (23)	10/13 (77)	
4	*S. schwetzi*	45/58 (78)	P = 0.007	12/13 (92)	1/13 (8)	Not computed
	*L. longipalpis*	41/42 (98)		0/1 (0)	1/1 (100)	
5	*S. schwetzi*	33/34 (97)	P = 1.000	0/1 (0)	1/1 (100)	Not computed
	*L. longipalpis*	25/25 (100)		-	-	
9	*S. schwetzi*	72/73 (99)	P = 0.447	1/1 (100)	0/1 (0)	Not computed
	*L. longipalpis*	24/25 (96)		0/1 (0)	1/1 (100)	

### Effect of decreased temperature on the PM and the development of *L. donovani*

**Figure 4:**
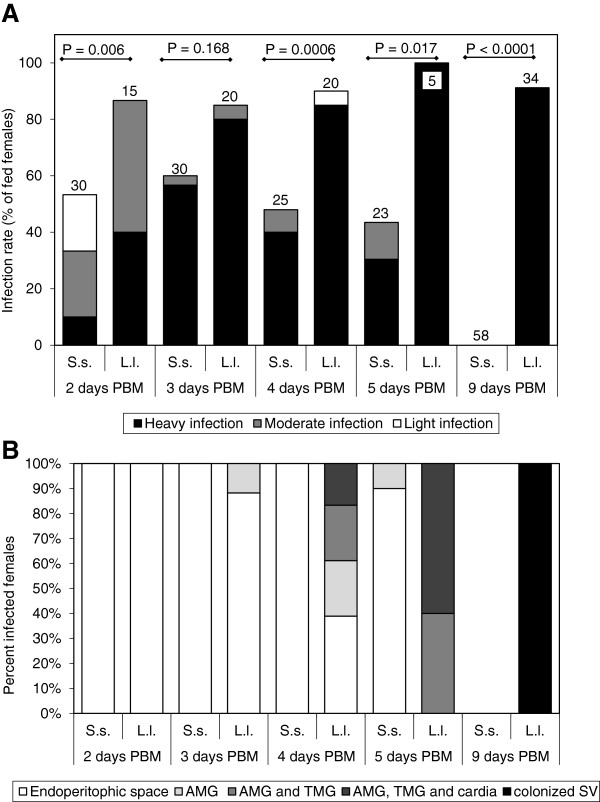
**Development of *L. donovani *in sand flies at 21°C. A)** Rates and intensities of infections in *Sergentomyia schwetzi* (S.s.) and *Lutzomyia longipalpis* (L.l.). Numbers of dissected females are shown above bars. Probability of differences was tested by Chi-square test. **B)** Location of *L. donovani* in infected *Sergentomyia schwetzi* (S.s.) and *Lutzomyia longipalpis* (L.l.). AMG, abdominal midgut; TMG, thoracic midgut; SV, stomodeal valve.

Lower temperature prolongs the duration of blood digestion in sand flies [23]. Therefore, we tested whether lowering the temperature to 21°C would result in enhanced development of *L. donovani* in *S. schwetzi*. At 21°C, the degradation of PM was delayed, it was present till day 4 and 5 PBM in *L. longipalpis* and *S. schwetzi*, respectively (Figure 4). The difference between vector species was significant, on day 5 PBM the PM was present in 78% of *S. schwetzi* and 0% of *L. longipalpis* (Chi-square = 10.957, d.f. = 1, P = 0.001).

In *S. schwetzi* delayed defecation resulted in higher infection rates on days 3 – 5 PBM and prolonged presence of *L. donovani* till day 5 PBM (Figure 2). However, from 22 positive females dissected on days 4 and 5 PBM, all but one had parasites still enclosed inside the PM (only in one female parasites were found free in the abdominal midgut). No infected *S. schwetzi* females were found on day 9 PBM. On the other hand, in *L. longipalpis* the lower temperature did not affect the infection rates, *L. donovani* developed well and on day 9 PBM all infected females showed heavy infections with colonization of the SV.

## Discussion

Demonstration of pathogen development under experimental conditions is one of the crucial parameters for vector incrimination [24]. Our observations clearly showed that *L. donovani, L. infantum* and *L. major* promastigotes did not develop late stage infections in *S. schwetzi.* They did not survive defecation of bloodmeal remnants and did not colonize the anterior midgut, which is the prerequisite for transmission by bite. Similar results were observed by Kaddu *et al.*[25]; *L. donovani* promastigotes produced only scanty parasitaemia in the abdominal midgut in three out of six *Sergentomyia* species without proper full-scale colonization of the thoracic midgut and cardia. Lawyer et al. [26] also described that Kenyan *S. schwetzi* does not support the development of *L. major*: for the first 48 hr, parasite development progressed but parasites were rarely seen after 48 hr and never after 90 hr PBM.

The mechanism of the resistance of *Sergentomyia* species to human *Leishmania* parasites is not clear and different hypotheses are plausible. Generally, there are several barriers in the sand fly midgut that must be overcome by the parasite to establish the infection: proteolytic enzymes produced during digestion of the bloodmeal, persistent peritrophic matrix and molecular characteristics of the midgut epithelium enabling or precluding the attachment of parasites. Parasites which do not overcome these midgut barriers are defecated with food remnants (for review see [27,28]).

Several authors have mentioned the fast digestion of the bloodmeal in *Sergentomyia*. Strelkova [29] and Reznik and Kuznecova [30] showed that destruction of erythrocytes in *S. arpaklensis* (corresponds to *S. sintoni* based on recent nomenclature) proceeded markedly faster in comparison with the *Phlebotomus* spp. These authors concluded that faster digestion was due to specialization of *Sergentomyia* for feeding on reptiles and digestion of nucleated erythrocytes [29]. Similarly, Lawyer *et al.*[26] observed faster digestion of the bloodmeal in *S. schwetzi* than in *P. duboscqi*.

However, the speed of bloodmeal digestion alone is not critical for *Leishmania* development in the vector. In our study, digested blood defecation by *S. schwetzi* females spanned over a significantly longer time period than in *L. longipalpis*. In addition, prolonged time of digestion induced by decreased temperature did not enhance the development of *L. donovani* in *S. schwetzi*; parasites were eliminated due to defecation after 5 days of development within the bloodmeal. Data indicated that the crucial aspect mediating the refractoriness of *Sergentomyia* was not the speed of digestion but the relative timing of defecation versus degradation of the PM.

Timing of disintegration of the PM in sand fly females may be important for the development of *Leishmania* promastigotes due to several reasons. Addition of exogenous chitinase to the bloodmeal blocked PM formation in *P. papatasi* which resulted in complete loss of *L. major* infections. These experiments showed that during the early phase of infections the PM can protect the parasites against the rapid diffusion of digestive enzymes [31].

Our previous study with *L. major* and *P. duboscqi* revealed that disintegration of the PM coincides with transformation of procyclic promastigotes to long nectomonads [32]. Broken PM ceases to form a mechanical barrier for parasites and enables the diffusion of signal molecules from the ectoperitrophic space to the vicinity of parasites and leads to their transformation. These signal molecules are probably salivary components ingested into the midgut [27], which are known to trigger parasite transformation *in vitro*[33,34]. While the procyclic promastigotes lack the ability to bind to midgut epithelium [35], highly motile nectomonad forms escape from the endoperitrophic space and bind to the midgut epithelium to avoid defecation together with bloodmeal remnants [27]. In this study, measurement of promastigotes in *L. longipalpis* on day 2 PBM revealed the presence of long nectomonads simultaneously with the disintegration of the PM. On the other hand, delayed transformation or elongation of *L. donovani* promastigotes was observed in *S. schwetzi* on day 2 PBM. Promastigotes mostly remained as procyclic forms probably due to lack of the signal molecules due to intact PM.

Persistence of the PM can influence *Leishmania* development in additional ways. A crucial parameter is the duration of the period between the degradation of the PM and defecation. On day 3 PBM, most *L. longipalpis* females had broken PMs but still retained blood remnants within the midgut as they did not defecate yet. Therefore, long nectomonads were free to leave the endoperitrophic space and attach to the *L. longipalpis* midgut wall. In *S. schwetzi*, the degradation of the PM was delayed often until defecation. Thus, there was either a very short time period between the degradation of the PM and defecation or the PM broke simultaneously with defecation. Therefore, promastigotes swimming freely in the ectoperitrophic space of *Sergentomyia* midgut were extremely rare. The persistence of the PM till the end of digestion was described also in *S. arpaklensis*[30,36] where it probably excluded transmission of *L. gymnodactyli* through the bite of *S. arpaklensis*[36].

Results of laboratory experiments suggest that findings of field studies should be interpreted with caution. Altogether, eleven species of *Sergentomyia* have been shown microscopically to carry *Leishmania* promastigotes in Kenya (reviewed by [37]) and Ethiopia [7]. However, these promastigotes were not characterized biochemically or genetically and are, therefore, not confirmed to be mammalian parasites. In addition, several *Sergentomyia* species were found to be PCR positive for DNA of human pathogenic *Leishmania* species: *L. major* DNA was found in *S. darlingi* in Mali [38], *S. garnhami* in Kenya [16] and in *S. sintoni* in Iran [39] while *L. donovani* DNA was detected in *S. babu* in Indian VL foci [40]. These results, however, do not mean that *Sergentomyia* spp. are involved in transmission of *L. major* or *L. donovani*. PCR positivity alone should not be used for incrimination of the sand fly (or other blood-sucking arthropod) as *Leishmania* vector; PCR of DNA does not detect whether parasites are viable and transformed to virulent metacyclic promastigotes. The early phase of *Leishmania* development in the vector is non-specific and promastigotes are able to develop in various bloodsucking arthropods, even in biting midges: *Leishmania* development was demonstrated in the *Culicoides nubeculosus* midgut until day 2 PBM, but a subsequent loss of parasites occurred, although a PCR-based assay indicated their presence for up to seven days [41].

In conclusion, our findings strongly advocate the need for vector competence confirmation by the precise microscopical observation of parasites in infected sand flies. In the case of human *Leishmania* species pathogenic to humans*,* it appears very important to detect heavy infections with metacyclic promastigotes colonizing the thoracic midgut and the stomodeal valve, which is a prerequisite for successful transmission by bite [28,42,43]. *Sergentomyia schwetzi*, together with other *Sergentomyia* species, well outnumbers the species of the genus *Phlebotomus* in VL foci in northern Ethiopia [8] where the burden of visceral leishmaniasis represents one of the most severe neglected tropical diseases (NTDs) in the region [44]. Newertheless, as we never found mature infections in *S. schwetzi* we conclude that this species, despite its overwhelming abundance in the Ethiopian VL foci, cannot serve there as the vector of *L. donovani*.

## Conclusions

Microscopical observations of *S. schwetzi* females infected with *L. donovani, L. infantum* and *L. major* clearly showed that these human pathogens are not able to develop late stage infections in this sand fly species*. Leishmania* did not survive defecation of bloodmeal remnants and did not colonize thoracic midgut and the stomodeal valve, which is the prerequisite for transmission by bite. Detailed study of females infected with *L. donovani* and maintained at different temperatures revealed that the crucial aspect mediating the refractoriness of *Sergentomyia* was the relative timing of defecation versus degradation of the PM. The PM remained intact till defection which probably also delayed the transformation of *L. donovani* promastigotes.

## Abbreviations

PM: Peritrophic matrix; VL: Visceral leishmaniasis; PBM: Post bloodmeal; AMG: Abdominal midgut; TMG: Thoracic midgut; SV: Stomodeal valve; S.s.: *Sergentomyia schwetzi*; L.l.: *Lutzomyia longipalpis*; P.d.: *Phlebotomus duboscqi*.

## Competing interests

The authors declare that they have no competing interests.

## Authors’ contributions

JS carried out the sand fly infections and dissections, morphometry of parasites, statistical analysis and drafted the manuscript. VD established the *S. schwetzi* colony and corresponded with the journal during submission of the manuscript. JV established the *S. schwetzi* colony and participated in the design of the study. VS participated in sand fly infections and dissections. AW conceived the study and helped to draft the manuscript. PV participated in the design of the study and helped to draft the manuscript. All authors read and approved the final manuscript.
